# Computed tomography osteoabsorptiometry-based investigation on subchondral bone plate alterations in sacroiliac joint dysfunction

**DOI:** 10.1038/s41598-021-88049-2

**Published:** 2021-04-21

**Authors:** A. Poilliot, T. Doyle, D. Kurosawa, M. Toranelli, M. Zhang, J. Zwirner, M. Müller-Gerbl, N. Hammer

**Affiliations:** 1 Department of Anatomy, University of Otago, 270 Great King Street, Dunedin, 9016 New Zealand; 2 Anatomical Institute, University of Basel, Basel, Switzerland; 3 University of Otago School of Medicine, Dunedin, New Zealand; 4 Department of Orthopaedic Surgery / Low Back Pain and Sacroiliac Joint Centre, JCHO Sendai Hospital, Sendai, Japan; 5 Department of Macroscopic and Clinical Anatomy, Medical University of Graz, Graz, Austria; 6 Department of Orthopaedic and Trauma Surgery, University of Leipzig, Leipzig, Germany; 7 Fraunhofer IWU, Dresden, Germany

**Keywords:** Bone, Skeleton, Preclinical research, Bone, Skeleton, Biomedical materials, Biomineralization, Bone quality and biomechanics, Bone

## Abstract

Sacroiliac joint dysfunction (SIJD) is an underappreciated source of back pain. Mineralization patterns of the sacroiliac (SIJ) subchondral bone plate (SCB) may reflect long-term adaptations to the loading of the joint. Mineralization densitograms of 27 SIJD patients and 39 controls, were obtained using CT osteoabsorptiometry. Hounsfield unit (HU) values of the SCB mineralization of superior, anterior and inferior regions on the iliac and sacral auricular surfaces were derived and statistically compared between SIJD-affected and control cohorts. Healthy controls showed higher HU values in the iliac; 868 ± 211 (superior), 825 ± 121 (anterior), 509 ± 114 (inferior), than in the sacral side; 541 ± 136 (superior), 618 ± 159 (anterior), 447 ± 91 (inferior), of all regions (*p* < 0.01). This was similar in SIJD; ilium 908 ± 170 (superior), 799 ± 166 (anterior), 560 ± 135 (inferior), sacrum 518 ± 150 (superior), 667 ± 151 (anterior), 524 ± 94 (inferior). In SIJD, no significant HU differences were found when comparing inferior sacral and iliac regions. Furthermore, HU values in the inferior sacral region were significantly higher when compared to the same region of the healthy controls (524 ± 94 vs. 447 ± 91, *p* < 0.01). Region mineralization correlated negatively with age (*p* < 0.01). SIJD-affected joints reflect a high mineralization of the sacral inferior region, suggesting increased SIJD-related mechanical stresses. Age-related SCB demineralization is present in all individuals, regardless of dysfunction.

## Introduction

Sacroiliac joint (SIJ) pain is often misdiagnosed when it is a functional disorder: (SIJ dysfunction: SIJD) related to hypo- or hypermobility, subluxation or misalignment^1^. SIJD typically does not result from acute inflammatory pathology, malformations nor injury, but may evolve from trauma^2,3^. 13–50% of patients with chronic lower back pain (LBP) suffer from SIJD^4^, but it is commonly underappreciated as a source of mechanical LBP^5^. Its clinical manifestations are diverse and mimic other musculoskeletal conditions making its diagnosis challenging^2^. Provocation tests show low specificity. Injections of local anesthetic are the current gold standard for definitive diagnosis of SIJD^6^, but remain controversial due to both false-positive and false-negative results^7^. Clinical radiography like computed tomography (CT) are not helpful for functional disorders and are either of limited value or invasive^8,9^. SPECT/CT is expected to detect the abnormal condition of the SIJ, but it is not widely used as its diagnostic value remains controversial^10^.

The subchondral bone (SCB) is known to adjust to long-term repetitive load transfer through an increase in bone mineralization which can be observed in CT-osteoabsorptiometry (CT-OAM) densitograms^11–14^. Given the effects of Wolff’s law, resulting in bone aligning to mechanical loading, the SCB adjusts to the load transfer though an increase in bone mineralization seen in CT-OAM^11,12,15^. CT-OAM provides colour-mapped densitograms using Hounsfield units (HU) to illustrate the bone mineralization of an articular surface^12–14,16–19^. Previous studies showed complete SIJ non-conformity and denser mineralization patterns on the iliac side^12^. High mineralization zones (≥ 700 HU) were found around the borders, apex and the corners of the auricular surfaces^12,20^. As asymmetrical gait and abnormal biomechanics are typically observed in SIJD patients, likely as a compensatory mechanism^21^, changes in bone mineralization patterns may reflect on the auricular surfaces caused by the abnormal loads instigated by SIJD. In unilaterally-affected SIJD, patients often load through their unaffected side for pain-relief leading to a decrease in muscle endurance and strength on the painful side which can affect the pelvic force-closure system^22,23^.

This given study aimed to compare the mineralization distribution patterns of the dysfunctional SCB in the superior, anterior and inferior regions of both the sacrum and the ilium, by comparing that of a cohort of SIJD patients with that of an age-matched healthy control cohort with no history of LBP. The following hypotheses were investigated:

1. SIJD patients display higher mineralization patterns compared to the healthy state.
2. Non-affected contralateral SIJs in patients with unilateral SIJD display similar mineralization patterns to healthy controls.
3. Mineralization patterns in the SIJ are different between patients with unilateral and bilateral SIJD.

## Materials and methods

### Patients and controls

**Figure 1:**
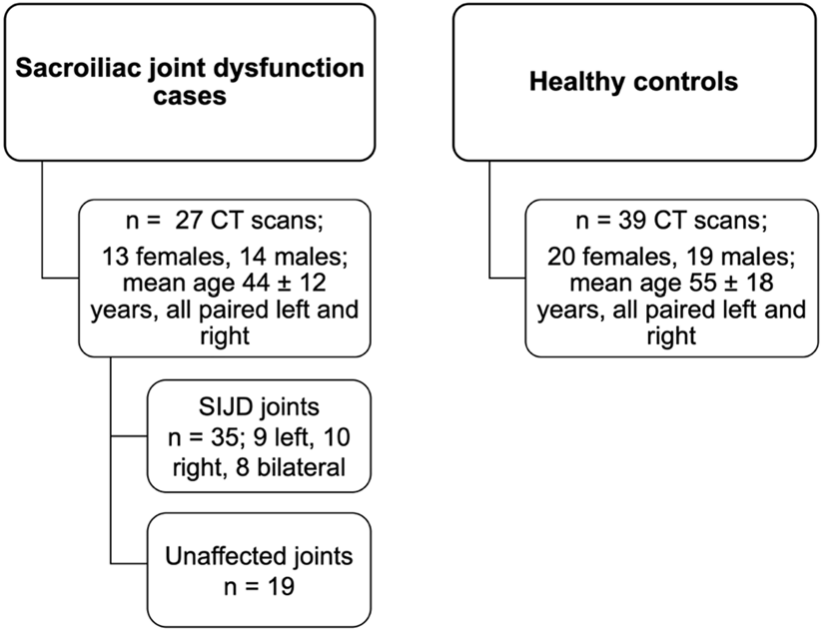
Flow chart of the patient computed tomography (CT) scans used for this study. Mean age ± standard deviation.* SIJD* sacroiliac joint dysfunction.

Twenty-seven patient cases diagnosed with unilateral or bilateral SIJD (13 females; 14 males; range 26 to 79 years) (Fig. 1) were collected between 2009 and 2018 in the JCHO Sendai Hospital, Sendai, Japan. Two cases were used in a previous study on sacroiliac joint arthrodesis for SIJD relief^24^. All patients identified the posterior superior iliac spine as the main pain area by using their index finger (one-finger test)^25^ and following thorough examination were considered to having SIJ pain. Definitive diagnosis of SIJD was confirmed by more than 70% pain relief at the SIJ region after SIJ local anesthetic injections under fluoroscopic guidance^26^. Patients with a history of infection, pathological conditions, tumors, cysts in the lumbopelvic area, recent lumbar spine and pelvic fractures, and seronegative spondylarthropathy were excluded. All included patients had a history of other injections including selective nerve root infiltration and/or lumbar disc nerve block that were negative. All patients were diagnosed as having severe chronic SIJD pain (for a minimum of 6 months) and reached the standard of indication for SIJ arthrodesis as ultima ratio: insufficient responsiveness to conservative treatments continued for longer than 6 months, difficulty in working, and/or marked restrictions of daily living due to recurrence of severe SIJ pain, even after undergoing repeated diagnostic/therapeutic injections and substantial physical therapy^24^. As a control cohort, 39 age-matched CT scans (20 females,19 males: range 21 to 82 years) acquired for clinical purposes to diagnose non-musculoskeletal pathologies, or to rule out injury related to acute trauma, were used. None of these cases had a current or past history of LBP, SIJ-related pathology or abnormalities on previous medical records. The Institute Review Board of JCHO Sendai Hospital, Sendai, Miyagi, Japan (no. 2019-1) approved the present study. Institutional approval was acquired for the use of patient datasets used in research studies for diagnostic and therapeutic purposes. Approval was granted on the grounds of existing datasets. Informed consent was obtained from all participants of this study. All methods were carried out in accordance with relevant guidelines and regulations.

### Computed tomography osteoabsorptiometry

Data sets for CT-OAM were derived from conventional CT (SOMATOM as64 open, Siemens, Munich, Germany; Aquilion one, Toshiba, Tokyo, Japan). Slices thicknesses averaged 1.25 mm for the healthy patient CT and ranged from 0.7 to 5.0 mm thickness for the scans of the cases with SIJD. CT-OAM was evaluated using a specific image analysis program (Analyze, v7.4, Biomedical Imaging Resources, Mayo Foundation, Rochester, NY, USA). According to a previous study^12^, the sacral and iliac sides of each SIJ were manually segmented within the CT datasets before the data were false color-coded and superimposed on the 3-dimensionally reconstructed ilia and sacra for anatomical localization of the mineral bone density creating a bone mineral density ‘densitogram’. The maximum intensity projection revealed the HU of each pixel to a depth of 3 mm^14^ and threshold values were chosen according to previous studies to be ≤ 200 to ≥ 1200 HU^12,13^.

### Analysis of densitogram patterns

**Figure 2:**
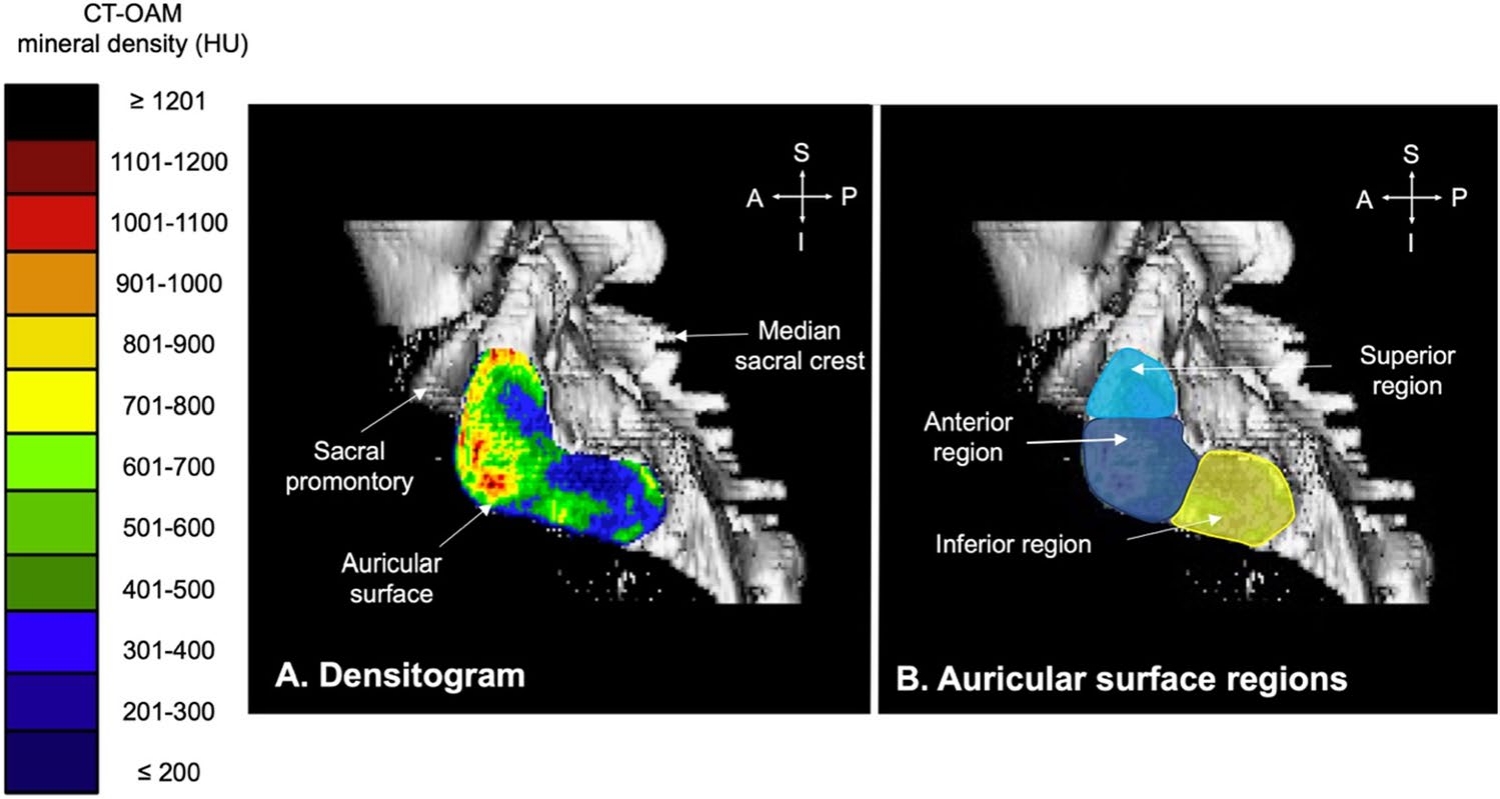
(**A**) Example of a sacral auricular surface densitogram with the Hounsfield unit (HU) scale on the left. (**B**) the same densitogram divided into the three regions, light blue: superior region, dark blue: anterior region and yellow: inferior region. The area of each region calculated the mean Hounsfield units’ value of that region. *CT-OAM* computed tomography osteoabsorptiometry, *A* anterior, *I* inferior, *P* posterior, *S* superior.

The mineral density pattern of the iliac and sacral sides was evaluated based on the mean HU values of the regions on the densitogram for each dataset (Fig. 2A). The auricular surfaces were subdivided into three regions: superior, anterior and inferior (Fig. 2B). These were defined as being three sections of equal size with the anterior region incorporating the apex of the auricular surface. The size of the tool was based on the size of the auricular surface of the specimen. Calculation of the mean HU value for each region was computed using non-calibrated CT grey values, obtained from a clinical-type scanner. These values were subsequently statistically compared between the different groups.

For statistical analyses GraphPad Prism (version 8, San Diego, CA, USA) was used. Statistical significance was defined at the 5% (*p* ≤ 0.05) level. Outlier values were identified using Microsoft Excel (version 15.38, Redmond, WA, USA) and removed from the data. These were values (n) outside the range of the upper and lower bounds of 1.5 × the interquartile range (IQR): (n < 1st quartile − (1.5 × IQR); n > 3rd quartile + (1.5 × IQR)). Gaussian distribution was first assessed using a Shapiro–Wilk test. Depending on the distribution, a one-way ANOVA or a Kruskal–Wallis test with Dunn’s post-hoc correction was undertaken for the multiple assessment of the data between the three regions. Mean HU values were reported ± standard deviation. Age correlations with mean HU values in the three regions between sexes, sides and within the bone were assessed using a two-tailed Spearman r test for non-parametric data or a two-tailed Pearson r test for parametric data. Correlation were defined as follows: strong ≥ 0.7, moderate 0.7 > r ≥ 0.5, weak 0.5 > r ≥ 0.3.

## Results

### Mineralization on the iliac side was consistently higher when compared to the sacral side except for the inferior region in SIJD

**Figure 3:**
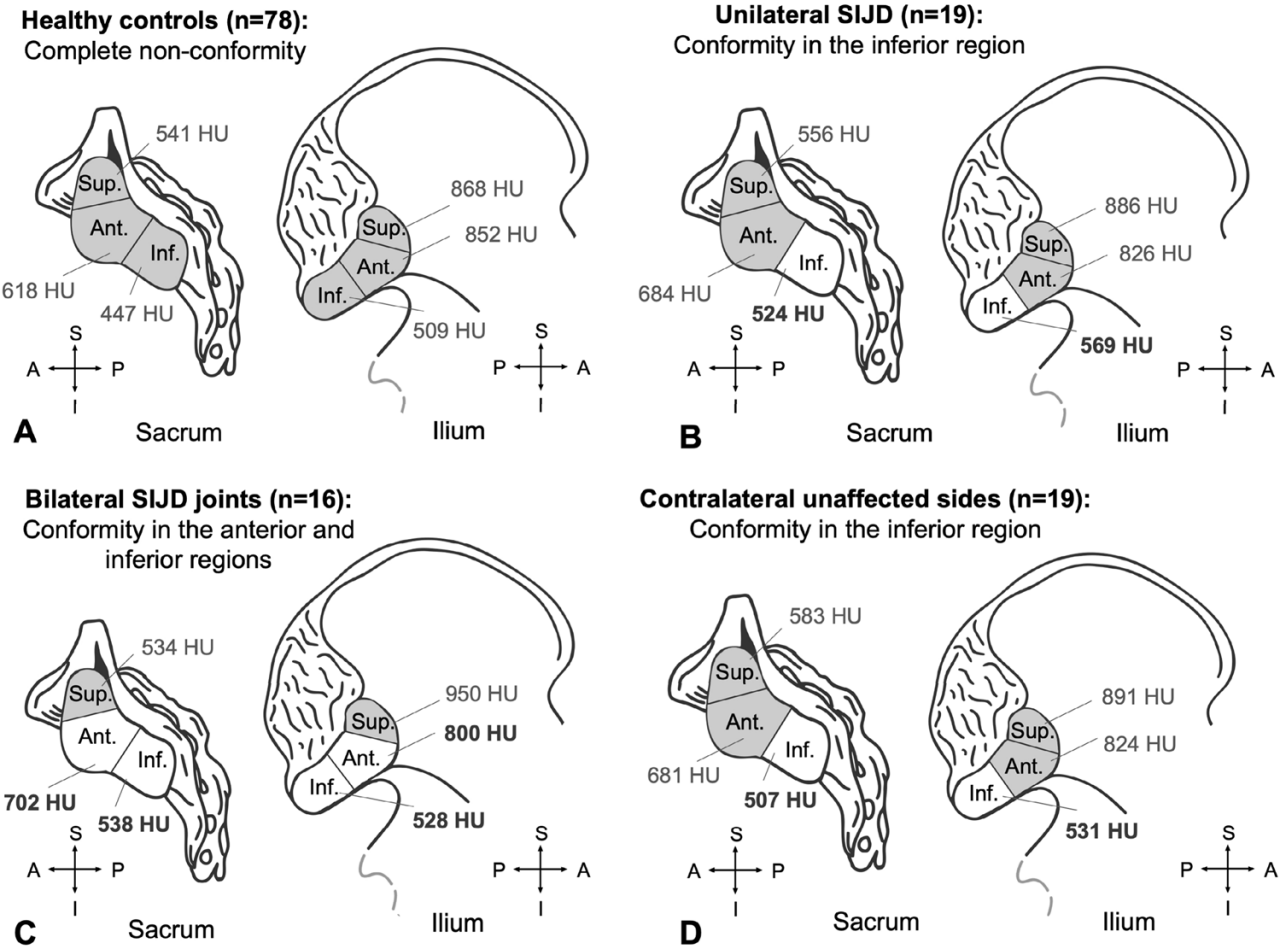
Conformity comparison between the sacral and iliac mean Hounsfield unit (HU) values in: (**A**) controls, (**B**), unilateral-SIJD, (**C**) bilaterally SIJD-affected cohort, (**D**) contralateral unaffected cohort. Shading represents non-conformity (significance at *p* < 0.05), *Sup.* superior region, *Ant.* anterior region, *Inf.* inferior region, *SIJD* sacroiliac joint dysfunction, *A* anterior, *I* inferior, *P* posterior, *S* superior.

In the SIJ of healthy controls, the extent of mineralization averaged for each region was consistently higher on the iliac side higher than the corresponding sacral region (*p* < 0.03; Fig. 3A). These values averaged 868 ± 211 HU (superior region, ilium), 541 ± 136 HU (superior region, sacrum), 825 ± 121 HU (anterior region, ilium), 618 ± 159 HU (anterior region, sacrum) and 509 ± 114 HU (inferior region, ilium), 447 ± 91 HU (inferior region, sacrum), respectively. In the SIJD cases, the same pattern was found (Fig. 3B,C). These values un-pooled averaged 908 ± 170 HU (superior region, ilium), 518 ± 150 HU (superior region, sacrum), 799 ± 166 HU (anterior region, ilium), 667 ± 151 HU (anterior region, sacrum) and 560 ± 135 HU (inferior region, ilium), 524 ± 94 HU (inferior region, sacrum).

**Figure 4:**
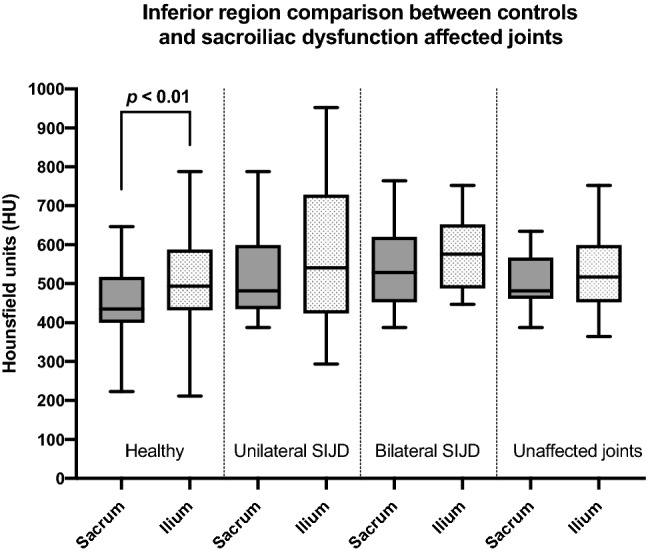
Box plot representing the mean Hounsfield unit values (HU) of the inferior sacral and iliac regions of healthy and sacroiliac dysfunction (SIJD) cohorts. The outlines of the boxes indicate the 25- and 75-percentile, the solid black horizontal line, the median. Whiskers indicate the minima and maxima. The dotted lines separate the cohorts in the table.

In the joints affected by unilateral SIJD and the contralateral unaffected sides, both the superior and the anterior regions of the ilium showed significantly higher HU values than the same regions of the sacrum (*p* < 0.05; Fig. 3B,D). In all SIJD cases, mean HU values of the inferior sacral region showed no significant differences when compared to the HU values of inferior region of the ilium (Fig. 4). In bilateral SIJD cases, HU values in both the anterior region and inferior region were high, and there were no significant differences when comparing the same regions of the ilium (*p* > 0.06; Fig. 3C). No significant differences in mean HU values were found between sexes (*p* > 0.6) nor sides (*p* > 0.1). See Supplementary Table S1 for all data.

### Differences in subchondral bone density exist in the inferior sacral region between SIJD cases and healthy controls

**Figure 5:**
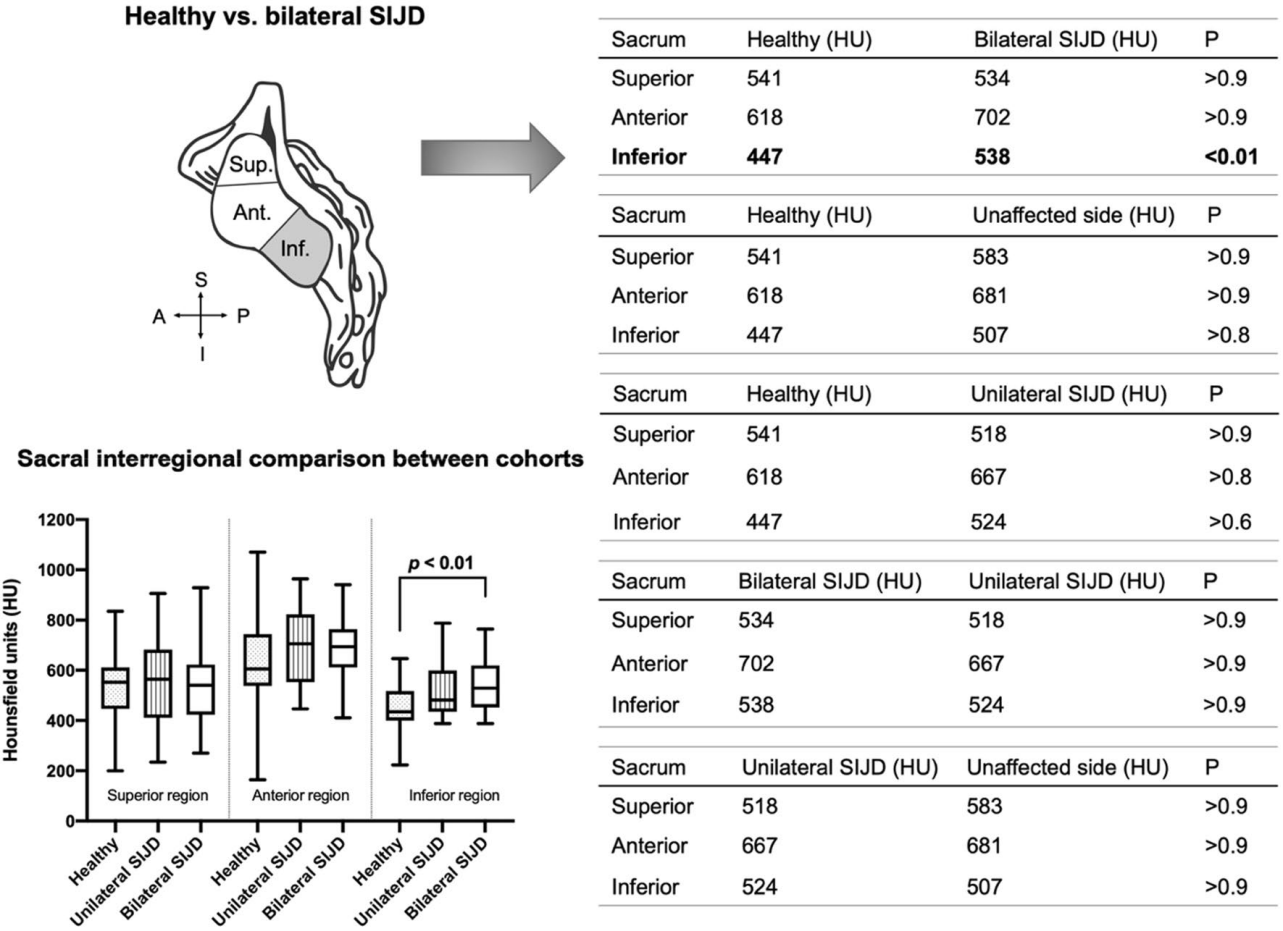
Box plot and table representing the mean Hounsfield unit values (HU) of all regions of the sacral side in the healthy and sacroiliac dysfunction (SIJD) cohorts. The outlines of the boxes indicate the 25- and 75-percentile, the solid black horizontal line, the median. Whiskers indicate the minima and maxima. The dotted lines separate the three regions in the table. Shading in auricular surface represents significant difference in mean Hounsfield units, *Sup.* superior region, *Ant.* anterior region, *Inf.* inferior region, *SIJD* sacroiliac joint dysfunction, *A* anterior, *I* inferior, *P* posterior, *S* superior.

When comparing the mean HU values of the sacrum between all SIJD cases and healthy controls, SIJD showed significantly higher HU values in the inferior sacral region (*p* < 0.01). Regarding the three types of comparison as follows; healthy versus unaffected joint with contralateral SIJD, and the unilateral and bilateral SIJD cases, there were no significant differences in any regions on either bone (Fig. 5). When comparing the healthy and unilateral SIJD joints, mean HU values of the inferior region of the sacrum in unilateral SIJD was higher than healthy controls but, it was not statistically significant. Only when comparing the inferior region of the sacrum between healthy and bilateral SIJD were the mean HU values significantly different (*p* < 0.01; Fig. 5).

### Loss of subchondral bone density is related to age, pronounced on the iliac side in healthy controls and on the sacral and iliac side in SIJD

**Figure 6:**
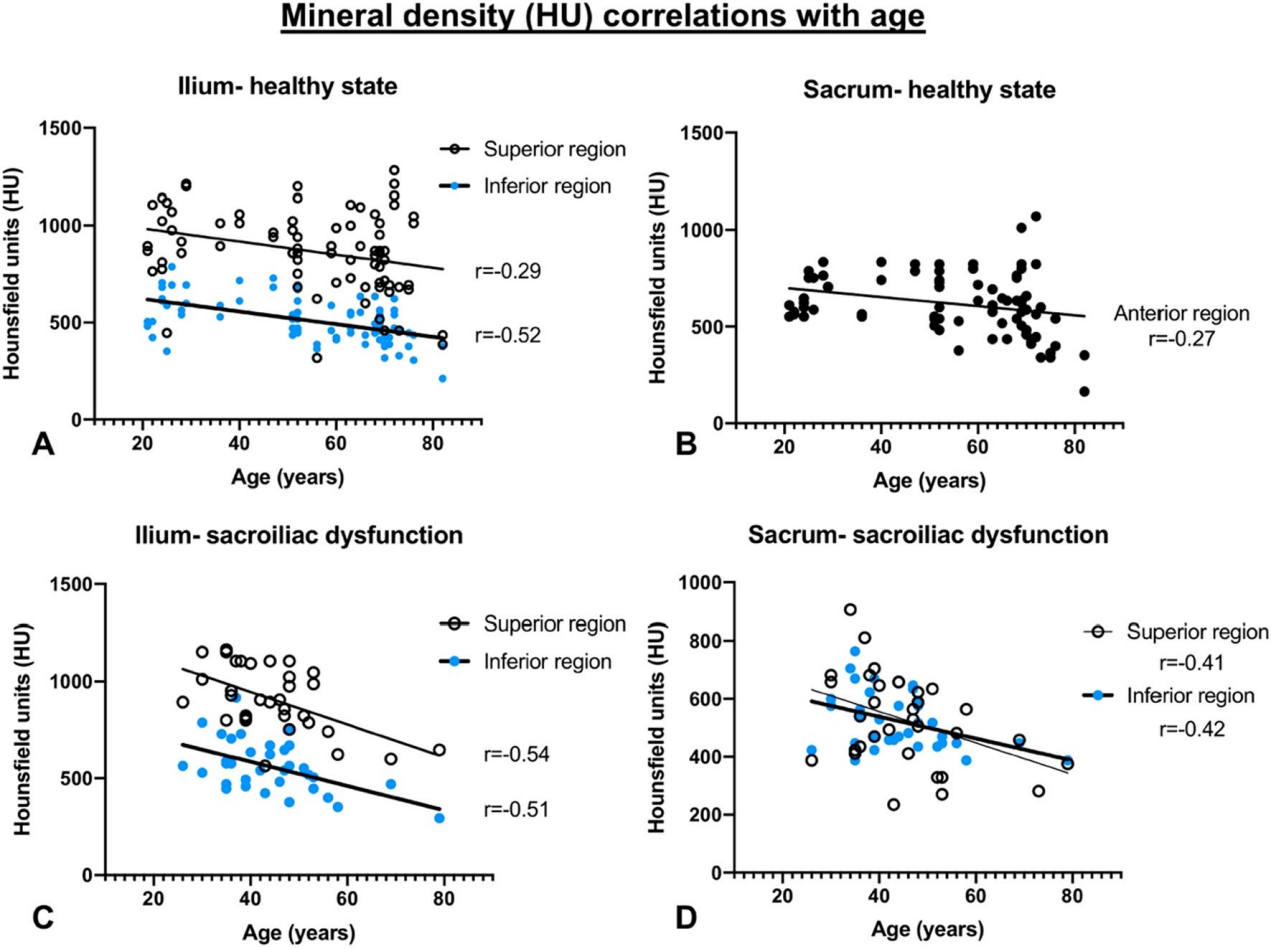
Significant age-correlations in healthy and sacroiliac dysfunction (SIJD) affected joints. (**A**) Healthy ilium, (**B**) Healthy sacrum, (**C**) SIJD-affected ilium, (**D**) SIJD affected sacrum.

In the healthy participants, correlation by age revealed an evident but weak to moderate negative correlation in the superior and inferior regions of the ilium (*p* < 0.01, r =  − 0.29, r^2^ = 0.08 superior; *p* < 0.01, r =  − 0.52, r^2^ = 0.27 inferior; Fig. 6A). The sacral side showed a weak negative correlation with age in the anterior region (*p* < 0.02, r =  − 0.27, r^2^ = 0.07; Fig. 6B). In SIJD, a weak to moderate negative age correlation on both bones in the superior (iliac: *p* < 0.01, r =  − 0.54, r^2^ = 0.30; sacral: *p* < 0.02, r =  − 0.41, r^2^ = 0.17) and inferior regions (iliac: *p* < 0.01, r =  − 0.51, r^2^ = 0.26; sacral: *p* < 0.01, r =  − 0.42, r^2^ = 0.17) (Fig. 6C,D). In the SIJD-unaffected joints with contralateral SIJD, age only moderately correlated in the sacral superior region (*p* < 0.02; r =  − 0.52; r^2^ = 0.27).

## Discussion

This given study quantified subchondral bone mineralization in the sacroiliac joint using CT-OAM in two patient cohorts: healthy SIJ controls and SIJD sufferers. It provides first insights into morpho-mechanic differences related to SIJD and densitograms of the sacral and iliac SCB based on mean HU values in the superior, anterior and inferior regions of the SIJ auricular surfaces. The patterns presented here are likely the result of chronically recurring loading conditions of individuals with and without SIJD, which represent the biomechanical stresses applied to the SIJ.

In healthy SIJs, the sacral inferior region showed lower mineralization values than that of the ilium. In SIJD, values of the sacral inferior region tended to be higher and closer to those of the ilium. Particularly in bilateral SIJD, in addition to the sacral inferior region, the anterior region HU values were higher. These are the most specific mineralization patterns of SIJD-affected joints. Thus, our first hypothesis; dysfunctional SIJs display higher mineralization patterns compared to the healthy state, can be confirmed in the inferior sacral region. The dysfunctional joint might be stressed in the sacral inferior region or, vice versa, stresses exerted to the inferior portion may be causative for SIJD. Anatomically, the posterior SIJ forms a syndesmosis. The anterior auricular region, however, consists of synovial tissues and a joint capsule like other typical synovial joints^27^. In an early stage of sacroiliitis, inflammation is often detected in the inferior portion of the joint^28^. Because the inferior portion of the joint is rich in synovial tissues, this indicates that the inferior region could be more mobile than the superior region. A recent study revealed that the upper portion of the SIJ was stressed in stance phase and the inferior portion was stressed in swing phase of bipedal walking^29^. The superior portion may work primarily as a shock-absorber and the inferior portion as a ‘slider’ for smooth bipedal walking. In cases of dysfunction, abnormal sliding of the joint surface may cause increased stresses in the inferior SIJ region. The sacral cartilage is thick originally and would contribute to stress-reduction of the sacral SCB in stance phase, but, in the inferior sacral region, the cartilage is thinner^30^. Therefore, in SIJD, the alterations in density of the inferior SCB could result from abnormal moving of the joint.

Furthermore, SIJ ligaments contribute to pelvic stability^31–33^ and can cause LBP^34,35^. In non-painful pelvic biomechanics, the pelvic musculature and surrounding soft tissues optimize the load-transmission through the cortical shell^36^. Increased mineralization in the inferior region may reflect impaired force-transmissions via the pelvic musculature and ligaments, perhaps laxity-related^37^. This may be the influence of SIJD, or it may reflect a ligamentous dysfunction elsewhere around the kinematic chain involving the inferior portions of the interosseous, posterior and the long SIJ ligaments, as well as the proximal parts of the sacrotuberous and sacrospinous ligaments^27,31^. Furthermore, sacral asymmetry had a higher prevalence in SIJD cases which might contribute to asymmetrical loading throughout life and might subsequently affect overall mineralization^38^.

No differences were found in any region on either bone when comparing the controls with the unaffected side of patients with unilateral SIJD, suggesting that SCB-mineralization is unaltered by preferential loading to the unaffected side^22,23^. When subjected to bilateral pain, it seems impossible to relieve the pain through compensatory mechanisms of a contralateral unaffected side, therefore, it is possible that both sides succumb to an increase in loads, specifically in the sacral inferior region, and could cause the increase in mineralization in this region. Thus, our second hypothesis; non-affected SIJs in patients with SIJD display similar mineralization patterns to healthy controls, can be approved. These findings could also suggest inflammation of tissues within the SIJ. This hypothesis has previously been rejected as inflammation and subsequent osteoblastic activity within the joint, would be observed on CT or bone-scans similar to sacroiliitis and ankylosing spondylitis, which has not been reported^8,9^. However, Maigne et al.^8^ reported an increased bone uptake of the subchondral lamella in the painful side of patients with SIJD, when using bone-scans. Although their results had poor sensitivity, it was suggested that this bony increase could reflect changes without an acute inflammatory episode^8^. In this case, the mineralization increases of the auricular surface may reflect low-grade inflammation of tissues which locally, may cause bone formation as seen in cases of aseptic loosening in hip arthroplasty^39^.

No significant change in patterns were found between patients with unilateral or bilateral SIJ pain, thus our third hypothesis; different mineralization patterns in the SIJ would be observed when comparing unilateral SIJD to bilateral SIJD joints, is rejected. Mineralization-induced density remained unaffected by altered biomechanics caused by unilateral pain, but it is impossible to know how many of the patients realistically exhibited altered loading in response to SIJD. Mean SCB density values were neither sex- nor side-dependent. Therefore, SCB mineralization is not related to side- nor sex influences in the loading of the posterior pelvis, occurring as a consequence of a different osteoligamentous alignment^31,33,40^. In all cohorts, negative age correlations were found when comparing the density values of each region, especially in the superior and inferior regions reflecting the osteopenia the bone succumbs to with time, caused by age-related changes in osteoanabolic metabolism^41^.

Regarding the limitations of the study, healthy scans were different patients to the SIJD cohorts; therefore, this comparison did not take into account population differences and potential variables between the two cohorts except, age, sex and SIJ pathologies. Size differences in the regions may have also been an influencing factor of the results as the regions were based on the size and shape of the auricular surface which would have been different for each individual. Furthermore, regional analysis may also have included some pixels outside of the auricular surfaces (HU value of 0), which may have caused an underestimation of the regional HU mean. In addition, the manual isolation of the three regions was performed by only one author. Therefore, the reproducibility of outlining the exact same regions was not tested. However, the regions were defined as being three sections of equal size with the anterior region incorporating the apex of the auricular surface. These are easily reproducible on each specimen regardless of the shape of the auricular surface.

## Conclusions

Joints with SIJD reflect increased stresses in the inferior portion of the SIJ, resulting from mechanically induced increases in density. Interregional comparisons reflect mineralization differences between healthy controls and SIJD. Subchondral bone mineralization is independent of sexes and sides but shows age-related morpho-mechanical alterations.

## Supplementary Information

Supplementary Information.

## Data Availability

The data acquired in the course of this study are available from the corresponding author on request.
