# Publisher Correction: Computed tomography osteoabsorptiometry-based investigation on subchondral bone plate alterations in sacroiliac joint dysfunction

**DOI:** 10.1038/s41598-021-95570-x

**Published:** 2021-08-02

**Authors:** A. Poilliot, T. Doyle, D. Kurosawa, M. Toranelli, M. Zhang, J. Zwirner, M. Müller-Gerbl, N. Hammer

**Affiliations:** 1 Department of Anatomy, University of Otago, 270 Great King Street, Dunedin, 9016 New Zealand; 2 Anatomical Institute, University of Basel, Basel, Switzerland; 3 University of Otago School of Medicine, Dunedin, New Zealand; 4 Department of Orthopaedic Surgery/Low Back Pain and Sacroiliac Joint Centre, JCHO Sendai Hospital, Sendai, Japan; 5 Department of Macroscopic and Clinical Anatomy, Medical University of Graz, Graz, Austria; 6 Department of Orthopaedic and Trauma Surgery, University of Leipzig, Leipzig, Germany; 7 Fraunhofer IWU, Dresden, Germany

Correction to: *Scientific Reports* https://doi.org/10.1038/s41598-021-88049-2, published online 21 April 2021

In the original version of this Article M. Müller-Gerbl was incorrectly affiliated with ‘Department of Orthopaedic Surgery/Low Back Pain and Sacroiliac Joint Centre, JCHO Sendai Hospital, Sendai, Japan’. The correct affiliation is listed below.

Anatomical Institute, University of Basel, Basel, Switzerland.

In addition, N. Hammer was omitted as a corresponding author. Correspondence and request for materials should also be addressed to niels.hammer@medunigraz.at.

The original Article and accompanying Supplementary Information file have been corrected.

