# Supplementary material for: Computed tomography osteoabsorptiometry-based investigation on subchondral bone plate alterations in sacroiliac joint dysfunction
Source: Sci Rep. 2021 Apr 21;11:8652. doi: 10.1038/s41598-021-88049-2 (PMC8060288; doi:10.1038/s41598-021-88049-2)
Supplement: Supplementary file 1 — Supplementary Information. [file 41598_2021_88049_MOESM1_ESM.pdf]

Computed tomography osteoabsorptiometry-based investigation on subchondral bone plate alterations in sacroiliac joint dysfunction

Poilliot A.[1], Doyle T.[2] Kurosawa D.[3], Toranelli M.[4], Zhang M.[1], Zwirner J.[1] Müller-Gerbl M.[4] & Hammer N.[5-7]

1 Department of Anatomy, University of Otago, Dunedin, New Zealand
2 University of Otago School of Medicine, Dunedin, New Zealand
3 Department of Orthopaedic Surgery / Low Back Pain and Sacroiliac Joint Centre, JCHO Sendai Hospital, Sendai, Japan
4 Anatomical Institute, University of Basel, Basel, Switzerland
5 Department of Macroscopic and Clinical Anatomy, Medical University of Graz, Graz, Austria
6 Department of Orthopaedic and Trauma Surgery, University of Leipzig, Leipzig, Germany
7 Fraunhofer IWU, Dresden, Germany

Corresponding authorship:

Amélie Poilliot, MSc, BSc (Hons)- ajpoilliot@outlook.com
270 Great King Street, Dunedin 9016, New Zealand
Tel: +64 3-479 7362

Niels Hammer, Professor, MD, Dr. med. habil.- niels.hammer@medunigraz.at
Auenbruggerpl. 2, 8036 Graz, Austria
Tel: +43 316 385 71100

Terence Doyle, Professor, MD, PhD- Terry.Doyle@southerndhb.govt.nz
Daisuke Kurosawa, MD, d-kurorin@m3.dion.ne.jp
Mireille Toranelli - mireille.toranelli@unibas.ch
Ming Zhang, Associate Professor, MMed, PhD- ming.zhang@anatomy.otago.ac.nz
Johann Zwirner, MD, Dr. med.- medijo@gmx.de

Co-senior authorship:

Magdalena Müller-Gerbl, Professor, Dr. med. habil. - m.mueller-gerbl@unibas.ch
Niels Hammer, Professor, MD, Dr. med. habil. - nlshammer@googlemail.com, niels.hammer@medunigraz.at

Supplementary material

Category (n)	Bone	Regions (mean ± SD)			P values		
		Superior	Anterior	Inferior	Sup. vs ant.	Ant. vs inf.	Sup. vs. inf.
**Healthy participants (n=78)**	Sacrum	541 ± 136	618 ± 159	447 ± 91	**p < 0.01***	**p < 0.01***	**p < 0.01***
	Ilium	868 ± 211	825 ± 121	509 ± 114	p > 0.5	**p < 0.01***	**p < 0.01***
	p values	**p < 0.01***	**p < 0.01***	**p < 0.03***			
**SIJD joints (n=35)**	Sacrum	518 ± 150	667 ± 151	524 ± 94	**p < 0.01***	**p < 0.01***	p > 0.9
	Ilium	908 ± 170	799 ± 166	560 ± 135	**p < 0.03***	**p < 0.01***	**p < 0.01***
	p values	**p < 0.01***	**p < 0.01***	p > 0.9			
**Joints with unilateral SIJD (n=19)**	Sacrum	556 ± 171	684 ± 162	524 ± 114	**p < 0.04***	**p < 0.01***	p > 0.7
	Ilium	886 ± 212	826 ± 198	569 ± 183	p > 0.9	**p < 0.01***	**p < 0.01***
	p values	**p < 0.01***	**p < 0.05***	p > 0.8			
**Joints with bilateral SIJD (n=16)**	Sacrum	534 ± 159	702 ± 144	538 ± 103	**p < 0.01***	**p < 0.01***	p > 0.9
	Ilium	950 ± 128	800 ± 156	528 ± 84	**p < 0.01***	**p < 0.01***	**p < 0.01***
	p values	**p < 0.01***	p > 0.06	p > 0.4			
**Unaffected contralateral joints (in unilateral SIJD cases) (n=19)**	Sacrum	583 ±161	681 ± 126	507 ± 75	p > 0.06	**p < 0.01***	p > 0.2
	Ilium	891 ± 225	824 ± 185	531 ± 111	p > 0.6	**p < 0.01***	**p < 0.01***
	p values	**p < 0.01***	**p < 0.02***	p > 0.9			

Supplementary table S1: *Inter-regional and inter-sides comparisons of the Hounsfield unit (HU) values of the sacroiliac joint within the different categories. Statistical significance is highlighted with an asterisk (*).*

SD = standard deviation; Sup. = superior region, Ant.= anterior region, Inf.= inferior region, SIJD= sacroiliac joint dysfunction.
